# *Candida albicans* and *C. tropicalis* Isolates from the Expired Breathes of Captive Dolphins and Their Environments in an Aquarium

**DOI:** 10.4061/2010/349364

**Published:** 2010-12-22

**Authors:** Hideo Takahashi, Keiichi Ueda, Eiko Nakagawa Itano, Makio Yanagisawa, Yoshiteru Murata, Michiko Murata, Takashi Yaguchi, Masaru Murakami, Katsuhiko Kamei, Tomo Inomata, Hirokazu Miyahara, Ayako Sano, Senzo Uchida

**Affiliations:** ^1^Medical Mycology Research Center, Chiba University, 1-8-1, Inohana, Chuo-ku, Chiba, 260-8673, Japan; ^2^Aquatic Mammal Section, Okinawa Churaumi Aquarium, 424 Ishikawa, Motobu-cho,Kunigami-gun, Okinawa, 905-0206, Japan; ^3^Laboratory of Applied Immunology, Department of Pathological Sciences, Center of Biological Sciences, State University of Londrina, 86051-970 Londrina, PR, Brazil; ^4^Laboratory of Molecular Biology, Department of Veterinary Medicine, Azabu University School of Veterinary Medicine, 1-17-71, Fuchinobe, Chuo-ku, Sagamihara, Kanagawa, 252-5201, Japan; ^5^Laboratory of Experimental Animals, Department of Veterinary Medicine, Azabu University School of Veterinary Medicine, 1-17-71, Fuchinobe, Chuo-ku, Sagamihara, Kanagawa, 252-5201, Japan

## Abstract

Genotypes of *Candida* spp. isolated from exhalation of 20 dolphins, 11 water samples from captive pools, and 24 oral cavities of staff members in an aquarium using a combination of multiple drug resistance 1 gene (*MDR1*) and the internal transcribed spacer (ITS) 1 5.8s-ITS 2 regions of ribosomal RNA gene (*ITS rDNA*) sequences were studied. The holding ratios of the dolphins, captive pools, and staff members were 70, 90, and 29%, respectively. Isolated pathogenic yeast species common to the dolphins and environments were *Candida albicans* and *C. tropicalis*. Identical genotypes in both *Candida* spp. based on the combination of *MDR1* and *ITSrDNA* were found in some dolphins, between a dolphin and a staff, among dolphins and environments, and among environments. The results indicated the diffusion and exchange of pathogenic yeasts at the aquarium among dolphins and environments. The isolates at the aquarium showed higher rates of resistance to azole antifungals compared to reference isolates.

## 1. Introduction

Mycotic diseases in delphinoids sometimes cause fatal outcomes or difficulties for the cares of animals [1–3]. Lobomysosis caused by *Lacazia loboi* (formerly *Loboa loboi*) is listed as the most famous mycosis in dolphins and zoonotic mycosis [1–12]. Apart from lobomycosis, *Aspergillus* spp. [1–3, 12–15], *Candida albicans* and other *Candida* spp. [16–20], *Chladosporium *sp. [21] and *Chladophialophora bantiana *[3], *Cryptococcus neoformans* [18–20, 22–24], *Fusarium* spp. [25], *Sporothrix schenkii* [26], *Trichophyton* sp. [27], *Trichosporn* sp. [1–3], and zygomycetes, [28–30] which are common to human fungal infections, have also been documented as causative agents for pulmonary, disseminated and cutaneous fungal infections in the animals [1–3]. Highly pathogenic mycoses caused by *Coccidioides immitis* [31], *Histoplasma capsulatum* [32], and *Blastomyces dermatitidis* [33] have also been reported. 

Besides being highly pathogenic, the above fungal species were isolated from exhalation, although the findings do not support correlations between mycoses—fungal pneumonia and/or disseminations and these organisms [3, 34]. In fact, even in healthy dolphins, pathogenic fungal species were isolated from exhalation [35]. Most species of pathogenic fungi isolated from exhalation were environmental contaminants while *C. albicans* and other *Candida* spp. existed as normal fungal residents of mucous membranes [3, 35]. Furthermore, these human pathogenic yeast species were isolated from more than 70% of captive dolphins and environmental water samples [19]. 

According to Buck [19] and Dunn et al. [17], there is no transmission or diffusion of *Candida* spp. between dolphins and environments. However, there has been no investigation of the correlation between human pathogenic yeast isolates from dolphins and their keeping conditions, including captive pools and staff members, based on molecular biological studies.

The present study aims to investigate the fungal flora of pathogenic yeast species from the exhalation of dolphins, captive pools, staff members, and air in front of the dolphin show stage at the Churaumi Aquarium, Okinawa, Japan, to clarify correlations among the isolates from dolphins, captive environments and staff members using genotypes of multiple drug resistance 1 gene (*MDR1*) having adequate sites of diversity for strain identification [36] and the internal transcribed spacer (ITS) 1 5.8 s-ITS 2 regions of ribosomal RNA gene (*ITS rDNA*) sequences recommended as the bar-coding gene of pathogenic fungi for differentiation of species [37].

## 2. Materials and Methods

### 2.1. Dolphins

The fungal flora of exhalation in dolphins captive at the Churaumi Aquarium, Okinawa, Japan, were investigated. The investigations were carried out on 20 individuals in August 2006 as sample collections in the summer time, and February 2007 in the winter. The sampled dolphins were as follows: two bottlenose dolphins (*Trusiops truncatus*), six (five in 2007 because of death) Indo-Pacific bottlenose dolphins (*Tursiops aduncus*), one Pacific bottlenose dolphin (*Trusiops aduncus*), three dolphins of F1 offspring between bottlenose dolphins and Pacific bottlenose dolphins, two Pacific white-sided dolphins (*Lagenorhynchus obliquiens*), six false killer whales (*Pseudorca crassidens*), and one rough-toothed dolphin (*Steno bredanensis*). The sex, age or estimated age, and housing periods in the aquarium are shown in Table 1. The survey was performed with the permission of Churaumi Aquarium, Okinawa, Japan, with a perspective for animal welfare.

### 2.2. Isolation and Identification of Pathogenic Yeasts from Dolphins

Four exhalations from each animal were collected. Two potato dextrose agar plates supplemented with 100 mg/L of chloramphenicol (CPDA) and two CHROMagar Candida plates (Kanto Chemical Co. Ltd., Tokyo, Japan) were placed approximately 40 cm above the blowhole. One plate per an exhalation was used. The plates were cultured at 25°C for 1 week, and the sprouted yeast colonies were counted. Colonies were collected according to slight differences in color on CHROMagar Candida plates and in size on CPDA. The collected yeast colonies were cultured on potato dextrose agar slants at 35°C for 48 hours. Colonies having growth ability at 35°C were identified on the basis of color on the CHROMagar Candida plates and species-specific polymerase chain reaction (PCR) for detecting topoisomerase II gene (*Top II* PCR) [43]. When plural isolates from one animal having identical genotype based on *MDR1* and *ITS rDNA* sequences have existed, the isolate was treated as one isolate. Mycelial colonies that grew on the plates were ignored at the present study.

### 2.3. Captive Pools and Discharged Water to the Sea

Samples were collected from eight pools of the aquarium for dolphins, two for manatees (*Trichechus manatus*), and at a discharging point of all pools to the sea. The relationship among pools and the water system is shown in Table 2.

Pools 1, 2, 3, and 8 communicated with each another and are supplied, seawater directly; this is indicated as water group A. Pools 4, 5, 6, and 7 indicated as water group B, also communicate with each another, and are supplied by sea water, and overflowing water from a fish aquarium. Pools 9 and 10 are supplied by salty well water, indicated as water group C. Seawater in discharge point is indicated as water D.

Water for captive pools is taken 300 m from the shore and at a depth of 20 m. Water exchange by an overflow system works at 8, 4, and 24 times of the volume of water per day at water systems A, B, and C, respectively. A complete change of water is provided 2 or 3 times a week, and this is aided by a scrubbing brush and by using 12% concentration of hydrochloride solution. 

Pools 1, 2, and 3 are used for the dolphin show at least 4 times a day, and sometimes the splash sprays on the audience. A special exhibition of training of a dolphin with an artificial tail fin is held at pools 4 and 5 at least twice a day, and the touching of dolphins by registered visitors is allowed every weekend and on holidays. Pools 9 and 10 also have opportunities for registered visitors to feed the manatees. 

The inhabiting dolphin members are not fixed. They are placed depending on the programs of dolphin shows, health conditions, and affinities. In contrast, the housing of the manatees is fixed depending on the sex. The dolphins and manatees are nursed and treated by the same staff members. Foot-bathing tubs with hydrochloride are placed at each pool entrance.

### 2.4. Isolation of Pathogenic Yeasts from Water Samples

Five hundreds milliliter of water samples taken from the surface of the captive pools were filtered with a 0.22 *μ*m pore-sized filter. The filters used for filtration were washed with 5 mL of sterile saline. One milliliter of the saline was put on CPDA and CHROMagar Candida plates and cultured at 25°C for 7 days. Yeast-form colonies were picked up and maintained on PDA slants at room temperature and identified by *Top II* PCR [43]. Mycelial colonies that grew on the plates were ignored at the present study.

### 2.5. Oral Pathogenic Yeast Flora of Staff Members

Twenty-four staff members (11 men, 13 women, 20–50 years old) were studied under personal agreement with informed consent. The survey was performed with the permission of Churaumi Aquarium, Okinawa, Japan. The ethic committee at the Chiba University judged that the present study had no infringement.

A sterile cotton tip was placed on the center of the tongue for 1 minute with rolling movements. The cotton tip was soaked in 2 mL of sterile-distilled water and then stirred vigorously for 10 seconds. One hundred microliters of the water was spread on a CHROMagar Candida plate cultured at 35°C for up to 7 days in duplicate. The sprouted yeast colonies were picked up and maintained on PDA slants at room temperature and identified by *Top II* PCR [43].

### 2.6. Airborne Fungi during Dolphin Show

One hundred liters and/or 500 L air was collected with an air sampler (Gunze, Tokyo, Japan) using an agar strip containing CPDA during the 4 dolphin shows at 11:00, 13:30, 15:00, and 16:00 in February 2007. The agar strips were cultured at 25°C for 7 days. The yeast colonies were picked up and cultured on PDA slants at 35°C for 2 and 7 days if the colonies were identified.

### 2.7. DNA Extraction

Fungal DNA was extracted with a DEXPAT Kit (TaKaRa, Ohtsu, Japan), following the manufacturer's protocol with slight modification, from cultures incubated on PDA slants at 25°C for 48 to 96 hours. Approximately 100 *μ*L of fungal mass was transferred to a sterilized microtube (1.5 mL) and homogenized with 0.5 mL of DEXPAT solution with a plastic pestle. The mixture was incubated at 100°C for 10 minutes and centrifuged at 12,000 pm (13, 201 g) for 10 min. The supernatant was used as the DNA sample.

### 2.8. Multiple Drug Resistant Gene 1 (MDR 1) Sequencing

The DNA was amplified with primers described by Tavanti et al. [36]. The primer sets for *C. albicans* were MDR1_CAF (5′-TGT TGT GTT TCA CTT TAC CT-3′) and MDR1_CAR (5′-AGG AGC ACC AAA TAA TGG GA-3′), and those for *C. tropicalis* were MDR1_CTF (5′-TGT TGG CAT TCA CCC TTC CT-3′) and MDR1_CTR (5′-TGG AGC ACC AAA CAA TGG GA-3′). DNA extract at 2.5 *μ*L, a piece of Ready-to-Go beads (Amersham Pharmacia, Tokyo, Japan), 2.5 *μ*L of 10 pM of the above primers, and 17.5 *μ*L of distilled water were mixed. Amplification was performed for an initial denaturing step of 7 min at 94°C, 30 cycles of 1 min at 94°C for DNA denaturation, 90 seconds at 55°C for primer annealing, 90 sec at 72°C for primer extension, a final extension of 10 min at 72°C, and a 4°C soak. The amplified PCR product was confirmed by electrophoresis on 1.0% agarose in 1x/TBE buffer (0.04 M Tris-boric acid, 0.001 M EDTA [pH 8.0]) and ethidium bromide staining. The amplified product was purified with a PCR purification kit (QIAquick PCR Purification Kit, QIAGEN, Hilden, Germany) according to the manufacturer's instructions. Cycle sequencing was performed with BigDye fluorescent-labeled DyeDeoxy protocols (BigDye Terminator ver. 1.01; Amersham Pharmacia, Piscataway, NJ, USA) as follows: 15 sec at 96°C, 30 sec at 55°C, and 4 min at 60°C for 25 cycles, followed by a 4°C soak. All sequencing reactions were run on an automated DNA sequencer (3100, Applied Biosystems, Foster City, CA, USA) after purification by ethanol precipitation. DNA sequences were aligned by GENETEX-MAC genetic information processing software (Software Development Co., Ltd. Tokyo, Japan).

### 2.9. ITS rDNA Sequencing

We used the same reaction system as that used for *MDR1 *detection with a piece of Ready-to-Go beads and the primer set ITS-5 (5′-GGA AGT AAA AGT CGT AAC AAG G-3′), and ITS-4 (5′-TCC TCC GCT TAT TGA TAT GC-3′) [38]. Amplification was performed for an initial denaturing step of 4 minutes at 95°C, 30 cycles of 1 minute at 94°C for DNA denaturation, 90 seconds at 50–53°C for primer annealing depending on samples, 2 min at 72°C for primer extension, a final extension of 10 min at 72°C, and a 4°C soak. After confirming the amplified DNA by electrophoresis, being purified using a kit, it was labeled with the primers ITS-5, ITS-4, ITS-2 (5′-GCT GCG TTC TTC ATC GAT GC-3′) and ITS3 (5′-GCA TCG ATG AAG AAC GCA GC-3′) [38] as follows: 15 sec at 96°C, 30 sec at 50 to 55°C, and 4 min at 60°C for 25 cycles, followed by a 4°C soak, and sequenced. 

Because yeast is a diploid organism, analysis of *MDR 1* and *ITS rDNA *sequences from both *C. albicans* and *C. tropicalis* was based on the data set of only the variable bases or the apparently predominant peak used previously with *C. albicans* multilocus sequence typing. When showing almost equal peaks, the base was determined as a wobble base using a universal code; K: G+T, M: A+C, R: A+G, S: C+G, W: A+T, and Y: C+T [36, 39, 40].

### 2.10. Genotypings

The *MDR 1* and *ITS rDNA *sequences from both *C. albicans* and *C. tropicalis* were deposited in the GenBank *via *DDBJ (DNA database of Japan, Mishima, Shizuoka, Japan). The combined sequences of *MDR 1* and *ITS rDNA *sequences were analyzed by the Unweighted Pair Group Method with Arithmetic mean (UPGMA) using GENETYX-MAC ver. 13.0 (GENETYX Corporation, Tokyo, Japan) genetic information processing software and given a serial number of genotype.

### 2.11. Susceptibility Testing

Susceptibility tests were performed according to the broth microdilution-modified method of the CLSI M27-A3 standard [41, 42] accepted standard using RPMI 1640 medium (Sigma, Poole, UK) buffered to pH 7.0 with MOPS (Sigma) and serial concentrations of amphotericin B (AMPH-B), flucytosine (5-FC), itraconazole (ITZ), fluconazole (FLCZ), miconazole (MCZ), and micafungin (MCFG). The latter three antifungals were included even though the method was originally described for use with AMPH-B, 5-FC, and ITZ. The test was performed in 96-well round-bottomed plastic plates using 100 mL of RPMI 1640 medium with fungal cells and antifungal substances (Dry plate koubo you, Eiken Co. Ltd., Tokyo, Japan). Data were obtained from duplicate trials. The mean or the lower data were taken. Reading results and the evaluation of the susceptibility categorized as susceptible, doze-dependent susceptible, intermediate, resistant and nonsusceptible on 5-FC, ITZ, FLCZ, and MCFG were followed to CLSI guideline [41, 42]. The category for susceptibility extracted from the CLSI guideline was shown in Table 1.

Twelve references isolates each for *C. albicans* and *C. tropicalis* stored in our center were added as references for molecular biological studies and the susceptibility test to antifungal substances.

## 3. Results

### 3.1. Isolates from Dolphins

The pathogenic yeasts isolated from exhalation of dolphins were *C. albicans*, *C. tropicalis*, and *C. glabrata*. The total numbers of colonies and identified species are shown in Table 1. Fourteen out of 20 dolphins, corresponding to 70% of the animals, had some kinds of pathogenic yeast species. The holding rates of *C. albicans*, *C. tropicalis,* and *C. glabrata* were 40%, 30%, and 5%, respectively.

Except for dolphin number 1, the rest of the fungal-positive animals had the same species of *Candida* in investigations of both August 2006 and February 2007 (Table 2). The genotypes and susceptibility to antifungal drugs of the *C. glabrata* isolate are not shown in the present study because there was only one isolate throughout the study.

Mycelial fungal species were also obtained from dolphin samples although the number of colonies was 1 or 2 per animal. The filamentous fungal species isolated from the exhalation of dolphins were shown in Table 3.

### 3.2. Isolates from Water, Staff Members, and Air

Collected water samples from 8 out of 11 sites from the captive pools and draining place had *C. albicans* and/or *C. tropicalis* during the summer investigation while the winter investigation resulted in 4 of the 11 sites showing only *C. albicans* isolates. The total number of colonies was less than 5 regardless of the collecting time or place. Except for Pool No. 10 nursing the male manatees, all water sources were positive for pathogenic yeasts for at least one collection period corresponding to 90.9% (Table 4). 

*Candida albicans* from 1 man and 2 women, *C. dubliniensis* from 1 woman, *C. parapsilosis* from 1 man, and *Cryptococcus albidus* from 1 man and 1 woman were isolated from the oral samples of staff members, corresponding to 29.2% (7 out of 24 individuals). The number of colonies per plate was more than 100, regardless of the sample. Except for *C. albicans*, genotypes and susceptibility to antifungal drugs were not shown in the present study. 

Although mycelial fungal species were *Aspergillus niger*, *A. ochraceus*, *Cladosporium* sp., *Fusarium* sp., *Penicillium* sp., and *Trichoderma* sp. identified in the basis of morphology, there was no pathogenic yeast from the air samples collected in the front of the dolphin shows.

### 3.3. Consistent Fungal Species between Dolphins and Environments

The consistent fungal species through the dolphins, the water samples, and staff members were *C. albicans* and *C. tropicalis, *as shown in Tables 5 and 6, respectively. The plural isolates from an animal or a site at the same collection period were due to the size of the colony on CPDA and/or CHROMagar Candida, the color on CHROMagar Candida, and the genotype based on the combination of *MDR1* and *ITS rDNA*.

### 3.4. MDR 1 and ITS rDNA Sequences in C. albicans

The sequences of *MDR 1* in *C. albicans*, consisting of 645 bases, were divided into 16 genotypes with at least 98.8% identity, and those in *ITS rDNA*, consisting of at least of 447 bases, were divided into 12 genotypes with at least 99.5% identity. Twenty-four genotypes based on combined sequences of *MDR 1* and *ITS rDNA* among *C. albicans *isolates showed more than 99.2% identity. Although we tried to determine the ITS sequences on isolates IFM 55378, 55281, and 55298 many times, these sequences were impossible to complete, because of extremely overlapping sequence. The accession numbers of the genes and the genotypes were shown in Table 5.

Except for *C. albicans *isolates derived from the dolphin No. 11 having 2 different genotypes of *C. albicans* collected at the 2006 summer, there was no dolphin having different genotype simultaneously at the same collecting time. *Candida albicans *isolates from dolphin numbers 3, 4, and 13 showed different genotypes depending on the collecting seasons. Isolates IFM 55378 derived from the dolphin No. 9 and IFM 55281 from the dolphin No. 16 were treated as exceptions for genotyping analysis, because of lacking *ITS rDNA* sequences. *Candida albicans *isolates from the captive pools; numbers 1, 2, and 4 of the same collecting period showed different genotypes, except for the isolate IFM 55298 lacked the genotype of *ITS rDNA*.

The genotypes A, B, G, and H are common among *C. albicans* isolates from dolphins and environment. The genotype C is identical between a dolphin and a staff. There was no common genotype between the isolates from the aquarium and the references in *C. albicans*. There was no common genotype between the isolates from the aquarium and the references in *C. albicans*.

### 3.5. MDR 1 and ITS rDNA Sequences in C. tropicalis

The sequences of *MDR 1* in *C. tropicalis*, consisting of 645 bases, were divided into 11 genotypes with at least 97.5% identity, and those in *ITS rDNA*, consisted of at least 435 bases, were divided into 5 genotypes with at least 94.5% identity. Thirteen genotypes based on combined sequences of *MDR 1* and *ITS rDNA* among *C. tropicalis* isolates had more than 97.2% identity. In addition, the *ITS rDNA* sequence of isolate IFM 55379 derived from dolphin No. 10 was not determined and treated as an exception for genotype analysis because of extremely overlapping signals. The accession numbers of the genes and the genotypes were shown in Table 4.

*Candida tropicalis* isolates from dolphin No. 7 collected in the summer had 2 genotypes and in the winter had independent one. Isolates from dolphin No. 2 were identical irrespective of the collecting time while dolphin No. 14 had different clones depending on the collecting time.

The genotypes A and B are common among *C. tropicalis* isolates from dolphins and environment. In addition, the genotype A was detected not only in isolates at the aquarium but also in the reference ones.

### 3.6. Antifungal Susceptibility

The susceptibilities to antifungal agents were shown in Tables 5 and 6. No isolate showed resistance to AMPB among the *C. albicans* and *C. tropicalis* isolates from dolphins, environments, and reference. 

Three of 15 (20%) from dolphins, 1 of 12 (8.3%) from the environments, and 1 of 12 (8.3%) from the references in *C. albicans* isolates showed resistance to 5-FC while none of *C. tropicalis* isolates regardless of origins showed resistance to 5-FC.

Thirteen of 15 (86.7%) from dolphins, 7 of 12 (58.3%) from the environments, and 1 of 3 (33.3%) from the staffs showed resistance or dose-dependent susceptibilities to FLCZ; however there was no isolate that showed resistance to the compound in the reference *C. albicans* isolates. Eight of 10 (80%) from dolphins, 1 of 6 (16.7%) from the environments, and 3 of 12 (25%) from the reference *C. tropicalis* isolates showed resistance or dose-dependent susceptibilities to FLCZ.

Twelve of 15 (80.0%) from dolphins, 10 of 12 (83.3%) from the environments, and 1 of 3 (33.3%) from the staffs showed resistance susceptibilities to ITZ; however no isolate showed resistance to the compound in the reference *C. albicans* isolates. Eight of 10 (80%) from dolphins, 3 of 6 (50%) from the environments, and 6 of 12 (50.0%) from the reference *C. tropicalis* isolates showed resistance or dose-dependent susceptibilities to ITZ.

There was no correlation between resistance or dose-dependent susceptibilities to antifungal agents and the genotype of *MDR1* or *ITS rDNA* in either *C. albicans* or *C. tropicalis* isolates. In addition, one isolate derived from the captive pool no. 5 collected at the winter 2007 showed extremely resistant to MCFG as 16 *μ*g/mL. The susceptibilities to MCZ were listed as reference data.

## 4. Discussion

### 4.1. Isolating Rates for Pathogenic Yeasts from Dolphins

According to Buck et al., the holding rates of *Candida *spp. in free-ranging dolphins were as follows: *C. albicans*, 7.0%; *C. tropicalis*, 14.3%; and *Candida *sp., 14.3% [35]. At the present study, the holding rates of *Candida* spp. in dolphins and captive-pools were 70% and 90.9%, respectively, which were higher than those of free-ranging dolphins. Similarly, anther report by Buck et al. [35] demonstrated that the captive environments of dolphins showed a higher incidence, over 70%, in feces and pool waters of captive bottlenosed dolphins (*Tursiops truncatus*). It suggested that the data from various aquariums or institutions might vary depending on the nursing conditions and the climates of the aquarium. Further studies will confirm the average data of the holding ratio of pathogenic yeast species in captive dolphins and their nursing environments with considerations of age, sex, and physiological data.

### 4.2. Relationship between Fungal Exhalation and Health

The relationship between fungal exhalation phenomena from blowholes and health condition has not been confirmed [3, 19], although many veterinarians, animal-keepers, and nurses in aquariums in Japan consider the isolations of *Candida* spp. from exhalation as being indicative of illness or weakness in dolphins [34]. We agree that a small numbers of total colonies in *C. albicans*, *C. glabrata,* and *C. tropicalis* isolates might be attached as normal fungal residents of mucous membranes, as described by Buck in 1980 [19], however, we had a doubt on the negative correlation between large numbers of *Candida* spp. colonies and a predictive sign of weakened health or preillness. There were 4 dolphins that dead after August 2006; for example dolphin No. 5 died in August 21, 2006 by pneumonia and colitis, No. 18 in December 24, 2007, No. 14 in January 15, 2008, and No. 13 in April 3, 2008 by pneumonia with long-term treatments. Two out of 4 dolphins showed a large numbers of *Candida* spp. in the breath when sampled indicating the correlation between large numbers of *Candida* spp. colonies and a predictive sign of weakened health or preillness. On the other hand, dolphin No. 7 had 407 *C. tropicalis* colonies in the summer and none in the winter, dolphin No. 10 had 601 colonies in the summer and 428 colonies in the winter did not die, and the majority of live dolphins had low Candida spp. rates. We could not confirm the relationship between the number of *C. tropicalis* colonies and the health condition of dolphins from these findings.

Although some pathogenic mycelial fungal species such as *Aspergillus niger*, *Aspergillus* spp., *Phoma* sp., *Curvularia lunata*, *Aspergillus* sp.*, Schizophyllum commune*, *Aureobasidium pullulans*, and *Fusarium* sp. were isolated from exhalation from blowholes, there was no correlation on the health of dolphins. We should wait for the accumulation of data on the fungal flora from exhalation and body conditions including blood and other physiological examinations, for judging the existence of the correlation.

### 4.3. Seasonal Characteristics on the Isolates

Interestingly, water source-derived *C. tropicalis* isolates disappeared in the winter, suggesting that *C. tropicalis* might have some difficulty surviving in winter conditions, and even in subtropical areas. The average temperatures of the environment and captive pools in February were 19.2°C and 22.2°C, while those in August were 31.4°C and 28.8°C, respectively. It is considered that the differences in water temperature might be one of the factors for the existence of *C. tropicalis*. Future investigations may confirm this phenomenon.

### 4.4. Genotypes

Genotypes based on *MDR 1* seemed to be suitable for molecular epidemiological study in a confined area due to adequate sites of diversity [36, 39]. On the other hand, genotypes of *ITS rDNA* could be useful for the identification of some intraspecies diversity and strain differentiations [44], and could show correlations to geographic, regional, and/or host-dependent genotypes of pathogenic fungi determined by multiple gene analysis [45]. Furthermore, the combination of 2 genes; *MDR 1* and *ITS rDNA,* could indicate more detailed diversity of the genotypes of *Candida* spp. than those by *MDR 1* or *ITS rDNA* alone. Then we discussed on the distributions of genotypes for *C. albicans* and *C. tropicalis* isolated in the aquarium based on the combined genotypes of *MDR 1* and *ITS rDNA*. In the basis of these combinations, we could demonstrate the existences of coincident pathogenic yeast species and their genotypes in both *C. albicans* and *C. tropicalis* between or among dolphins, captive-pools and a staff member although Buck have denied the possibility that pathogenic yeasts are dispersed to other dolphins and environments [19]. Especially, the common genotype of *C. albicans* to both a dolphin and a staff member might be exchanged between them since the animal has been receiving medicine and surgical treatments from the staff member working as a veterinarian. 

Interestingly, the genotype of *C. albicans* isolated from dolphin No. 5 which suddenly died of bacterial colitis and pneumonia was detected in the isolate from dolphin No. 3 and captive pools numbers 1, 2, and 4. Those of *C. tropicalis* isolated from the same dolphin No. 5 have also been detected in the isolate from dolphin No. 10, from captive pools No. 5 and 6, and from drained seawater. The animal might be a dispersal source for both *C. albicans* and *C. tropicalis* to other animals as well as to the environment. 

The source of pathogenic yeasts might be related to the environmental water since common genotypes among the dolphins and pool water samples were found in *C. albicans* and *C. tropicalis* isolates. Isolates from captive pools free of dolphins had the same genotypes of *Candida* spp. isolates as the dolphins. Nevertheless, any systematic relationship of the water supply could not be found between pathogenic yeast species and captive pools or seawater. Exchanges of dolphins, common staff members for dolphins and manatees, and/or the influence of the audience might play roles in the dispersing, exchanging and introducing pathogenic yeasts. Further studies and detailed molecular profiles of the isolates may confirm the principle sources of the pathogenic yeasts.

The coincidence of genotypes in dolphins and in environmental isolates to the reference isolates of *C. tropicalis* suggested that such genotypes might be introduced from audiences or from sea water, and/or be very common in the world.

### 4.5. Attention for Sample Collection

Attention to plural isolates from the same animal at the same sampling period might be important. Although the differences in the colonies were slight with regard to size on CPDA or color on CHROMagar Candida, the clones showed different genotypes and/or susceptibilities to antifungal agents as detected in the *C. albicans* isolates IFM 55273 and IFM 55274 derived from dolphin No.11 isolated at the August 2006. Therefore, at least 2 or more colonies, depending on size and/or color, should be selected for identification, molecular biological analysis, and susceptibilities to antifungal drugs.

### 4.6. Risk to Be Audience

The fishy smell in the auditorium of the dolphin show indicates a possible spread of the breath, including pathogenic yeasts, to the audience, in spite of the fact that such pathogenic yeast isolates from air samples collected in the front of the dolphin shows were not detected. This suggests that the possibility of inhaling or being exposed to pathogenic *Candida* spp. from the exhalation of dolphins is relatively low. Nevertheless, it seems dangerous to approach the blowholes to a distance closer than 40 cm. For example, activities such as kissing or touching dolphins and, for pregnant women, swimming with dolphins should be approached with caution. The exact distance from the blowholes of dolphins from where it would be free of yeast-blow needs to be measured. Although there was no record of fungal infection caused by inhalation of the exhalation of dolphins, an immunocompromised person should be strongly urged to avoid such close contact with dolphins.

### 4.7. Characteristics on the Susceptibilities to Antifungal Drugs

The high ratio in isolations of pathogenic yeasts derived from the dolphins compared with the reference strains was the same as that in human oral fungal flora with HIV-infected or immunocompromised hosts [46, 47]. Frequent administrations of antibiotics and steroids might be one of the explanations, but data regarding the parameters concerning stress and immunosupression, defense mechanisms against microorganisms, and drug metabolisms in the animals, even in normal immune data or blood chemical profiles, are not yet sufficient for meaningful discussion, although the correlation between the occurrence of lobomycosis and immune status of the dolphins was reported in Floridan bottlenose dolphins [10, 48–51]. 

Furthermore, the higher incidences of resistance to azole-related antifungal agents in the isolates from dolphins and environments might be related to the sodium chloride in sea water, a speculation drawn from the correlations among resistance to chemical compounds, pathogenicity, and sodium chloride [52].

### 4.8. Correlation between the Genotype and Resistance to Antifungal Agents

According to Tavanti et al., *MDR 1* alone cannot define the relationship between genotypes and profiles of antifungal agents [36]. In the present study, no correlation was found between resistance or dose-dependents susceptibilities to azole-related antifungal agents and the genotypes of *MDR1* and/or *ITS rDNA* in either *C. albicans* or *C. tropicalis*, since an identical genotype based on *MDR1* and *ITS rDNA* sequences has shown different profiles of susceptibilities to azole-related antifungal agents, regardless of origin. The reference isolates, giving a higher ratio of resistant isolates, could not help in determining the specific genotypes based on the combination of *MDR1* and *ITS rDNA* sequences.

## 5. Conclusions

The detection of common genotypes on *Candida* spp. among dolphins, environments, and staff members pointed to the dissemination of pathogens at the aquarium. Thus, it seemed to be important to consider the effects on audiences from dolphins and the reverse relations for controls of zoonotic infections. In addition, the sequence of *MDR 1* showed adequate numbers of variations, indicating that the gene might be useful for molecular epidemiological studies.

## Figures and Tables

**Table 1:** Interpretive guidelines for *in vitro* susceptibility testing of *Candida* spp. extracted from CLSI guideline.

Antifungal agent	Susceptible	Susceptible-dosedependent	Intermediate	Resistant	Nonsusceptible	Reference
	(S)	(SSD)	(Ib)	(R)	(NS)*	
Amphotericin B	—	—	—	≧1	—	[41]
Flucytosine	≦4	—	8–16	≧32	—	[42]
Fluconazole	≦8	16–32	—	≧64	—	[42]
Itraconazole	≦0.125	0.25–0.5	—	≧1	—	[42]
Micafungin	≦2	—	—	—	>2	[42]

*;special term for echinocandin, and has as the same meaning as resistant.

**Table 2:** Dolphins and pathogenic yeasts isolates.

Dolphin no.	Name	Animal species	Remarks		Aug. 2006		Feb. 2007	
			Sex	age at feb. 2007	No. of	Fungal	No. of	Fungal
				(Death record)	colonies	species	colonies	species
1	Gon	FKW	F	Approx. 30	16	*C. tropicalis*	1	*C. albicans*
2	Sky	BD	M	7	12	*C. tropicalis*	59	*C. tropicalis*
3	Sami	IOBD	F	7	9	*C. albicans*	2	*C. albicans*
4	Fuji	BD	F	Approx. 36	1	*C. albicans*	1	*C. albicans*
5	Kana^D^	F1	F	9 (died at 21, Aug. 2006)	225	*C. tropicalis*	ND	ND
6	Oki	IOBD	F	Approx. 33	4	*C. glabrata*	6	*C. glabrata*
7	Okigon-4	FKW	F	Approx. 12	407	*C. tropicalis*	None	—
8	Kama-2	PWD	M	Unknown	None	—	None	—
9	Cony	F1	F	17	2	*C. albicans*	23	*C. albicans*
10	Chao	F1	M	11	601	*C. tropicalis*	428	*C. tropicalis*
11	Kuro	IOBD	M	Approx. 35	13	*C. albicans*	2	*C. albicans*
12	Okigon-1	FKW	M	Approx. 11	None	—	None	—
13	Larf ^D^	RTD	M	Unknown (died at 3, Apr. 2008)	2	*C. albicans*	26	*C. albicans*
14	Okigon-3^D^	FKW	F	Approx. 35 (died at 15, Jan. 2008)	291	*C. tropicalis*	2425	*C. tropicalis*
15	Muku	IOBD	M	Approx. 35	None	—	None	—
16	Dan	IOBD	M	Approx. 38	20	*C. albicans*	None	—
17	Poi	IOBD	M	Approx. 35	1	*C. albicans*	None	—
18	Chura^D^	FKW	F	6 (died at 24, Dec. 2007)	None	—	None	—
19	Momo	FKW	F	Approx. 4	None	—	None	—
20	Kama-1	PWD	F	Unknown	None	—	None	—

FKW: False killer whale (*Pseudorca crassidens*), BD: Bottlenose dolphin (*Tursiops truncatus*), IOBD: Indo-Pacific bottlenose dolphin (*Tursiops aduncus*), PWD: Pacific white-sided dolphin (*Lagenorhynchus obliquidens*), F1: F1 offspring between BD and IOBD, RTD: Rough-toothed dolphin (*Steno bredanensis*), Approx.: Approximately, *; total numbers of colonies obtained from 2 CHROMagar Candida and 2 CPDA plates. Dolphin number. 5 was died of enterocolitis and pneumonia, and numbers 13, 14 and 18 were of pneumonia caused by bacterial infections.

**Table 3:** Mycelial fungal species isolated from dolphins.

Dolphin no.	Name	fungal species (collected period)
1	Gon	Unidentified white filamentous fungi (S)
2	Sky	2 different colonies of unidentified white filamentous fungi (S)
3	Sami	*Phoma* sp. (W), *Curvularia lunata* (W), unidentified white filamentous fungi (W)
4	Fuji	*Aspergillus* sp. (S)
5	Kana	*Schizophyllum commune* (S), *Aureobasidium pullulans* (S)
6	Oki	None
7	Okigon-4	*Cladosporium cladosporioides* (W)
8	Kama-2	2 different colonies of unidentified white filamentous fungi (S)
9	Ciny	None
10	Chao	*Aspergillus* sp. (S)
11	Kuro	*Curvularia lunata* (S), unidentified white filamentous fungi (S)
12	Okigon-1	Unidentified white filamentous fungi (S)
13	Larf	Unidentified white filamentous fungi (W)
14	Okigon-3	*Fusarium* sp.
15	Muku	*Aspergillus* sp. (S), *Penicillium* sp. (W)
16	Dan	*Aspergillus niger*,(S) *Arthrinium phaeospermum* (S), unidentified white filamentous fungi (S)
17	Poi	3 different colonies of unidentified white filamentous fungi (S)
18	Chura	None
19	Momo	None
20	Kama-1	None

S: August 2006; W: February 2007.

**Table 4:** Pools and seawater.

				Isolate	
No.	Pool name	Remarks	Water system	Aug. 2006	Feb. 2007
1	H1	Adjunct to the main pool	A	*C. tropicalis*	*C. albicans*
2	H2	Adjunct to the main pool	A	*C. albicans*	None
				*C. tropicalis*	
3	Main pool*	Dolphin shows	A	*C. albicans*	None
				*C. tropicalis*	
4	Lagoon shallow	Dolphin show of training for artificial fin,	B	*C. albicans*	None
		Touching dolphins every weekend,			
		adjunct to lagoon main			
5	Lagoon main	Dolphin show of training for artificial fin	B	*C. tropicalis*	*C. albicans*
6	Lagoon H1	Adjunct to lagoon main	B	*C. tropicalis*	None
7	Lagoon H2	Adjunct to lagoon main	B	None	*C. albicans*
8	Studio	Independent pool from No. 1–7 supplied	A	None	*C. albicans*
		by as the same water system as pools No. 1–3.			
9	Manatee female	Indoor and apart from dolphin pools	C	*C. albicans*	None
10	Manatee male	Indoor and apart from dolphin pools	C	None	None
11	Sea water	Discharge point for all pool water	D	*C. albicans*	None
				*C. tropicalis*	

**Table 5:** Antifungal susceptibility and genotypes of C. *albicans* isolates.

		Susceptibility to antifungal drugs						MDR 1		ITSrRNA		Combined
IFM No.	Animal No.	AMPH-B	5-FC	FLCZ	ITZ	MCZ	MCFG	Accession No.	Genotype (1–16)	Accession No.	Genotype (I–XII)	Genotype (A–X)
Dolphin isolates												
55372	No. 1 (W)	0.5	<0.125	>64*	>8*	4	<0.03	AB379716	1	AB437006	V	A
55224	No. 3 (S)	0.5	<0.125	>64*	>8*	8	<0.03	AB379697	1	AB436989	VI	B
55374	No. 3 (W)	0.5	<0.125	>64*	>8*	2	<0.03	AB379717	1	AB437007	VI	B
55226	No. 4 (S)	0.25	0.25	32^+^	8*	2	<0.03	AB379699	16	AB436991	V	C
55376	No. 4 (W)	0.25	0.125	1	0.125	2	<0.03	AB379718	16	AB437008	V	C
55292	No. 5 (S)	0.5	<0.125	64*	8*	2	<0.03	AB379706	1	AB436997	VI	B
55267	No. 9 (S)	0.25	>64*	>64*	>8*	1	<0.03	AB379700	15	AB436992	VI	D
55378	No. 9 (W)	0.25	>64*	>64*	>8*	32	<0.03	AB379719	15	ND	ND	ND
55273	No. 11 (S)	0.25	0.125	>64*	>8*	4	<0.03	AB379701	15	AB436993	VI	D
55274	No. 11 (S)	0.5	<0.125	2	0.125	1	<0.03	AB379702	15	AB436994	V	E
55381	No. 11 (W)	0.25	<0.125	64*	2*	8	<0.03	AB379720	15	AB737009	XI	F
55276	No. 13 (S)	0.25	<0.125	>64*	>8*	4	<0.03	AB379703	7	AB436995	V	G
55382	No. 13 (W)	0.25	<0.125	64*	8*	4	<0.03	AB379721	7	AB437010	V	G
55281	No. 16 (S)	0.125	>64*	>64*	>8*	2	<0.03	AB379704	15	ND	ND	ND
55290	No. 17 (S)	0.5	<0.125	8	0.125	<0.06	<0.03	AB379705	1	AB436996	IX	H
Environmental isolates												

55384	Pool-No. 1 (A) (W)	0.5	<0.125	>64*	>8*	2	<0.03	AB379722	1	AB437011	VI	B
55385	Pool-No. 1 (A) (W)	0.5	<0.125	0.125	0.03	0.06	<0.03	AB379723	7	AB437012	V	G
55302	Pool-No. 2 (A) (S)	0.5	<0.125	>64*	>8*	2	<0.03	AB379710	1	AB737000	IX	H
55304	Pool-No. 2 (A) (S)	0.25	<0.125	64*	>8*	2	<0.03	AB379711	1	AB437001	VI	B
55298	Pool-No. 3 (A) (S)	0.5	<0.125	>64*	>8*	2	<0.03	AB379709	1	ND	ND	ND
55295	Pool-No. 4 (B) (S)	0.5	<0.125	4	>8*	2	<0.03	AB379707	1	AB436998	VI	B
55867	Pool-No. 4 (B) (S)	0.5	<0.125	4	2*	2	<0.03	AB379708	1	AB436999	IX	H
55388	Pool-No. 5 (B) (W)	0.25	<0.125	>64*	>8*	16	>16	AB379726	1	AB437015	V	A
55387	Pool-No. 7 (B) (W)	0.25	>64*	>64*	>8*	>32	<0.03	AB379725	15	AB437014	VIII	I
55386	Pool-No. 8 (A) (W)	0.25	<0.125	>64*	>8*	>32	<0.03	AB379724	1	AB437013	VIII	J
55871	Pool-No. 9 (C) (S)	0.5	<0.125	8	1*	2	<0.03	AB379712	15	AB707002	VIII	I
55319	Sea Water (D) (S)	0.25	<0.125	4	0.06	2	<0.03	AB379714	15	AB437004	VIII	I
Staff isolates												

55390	Staff-A	0.25	<0.125	>64*	>8*	8	<0.03	AB379727	16	AB437016	V	C
55392	Staff-B	0.5	<0.125	2	0.125	0.5	<0.03	AB379728	11	AB437017	V	K
55395	Staff-C	0.5	<0.125	0.5	0.03	2	<0.03	AB379729	5	AB437018	II	L
Reference isolates												

4953	Sputum (J)	0.25	<0.125	0.25	0.03	<0.06	<0.03	AB379730	9	AB437019	I	M
5633	Oral mucosa (J)	0.25	<0.125	0.25	0.03	0.06	<0.03	AB379731	13	AB437020	V	N
5713	Sputum (J)	0.25	<0.125	0.125	0.03	<0.06	<0.03	AB379732	10	AB437021	IV	O
40213	Blood (USA)	0.5	0.25	0.25	0.03	0.06	<0.03	AB379735	2	AB437024	V	P
=ATCC 90028												

41419	Sputum (J)	0.25	<0.125	0.25	0.03	0.06	<0.03	AB379736	4	AB437025	X	Q
49764	Oral mucosa (J)	0.5	<0.125	0.25	0.03	0.06	<0.03	AB379741	4	AB437029	V	R
49765	Oral mucosa (J)	0.5	>64*	8	0.06	0.06	<0.03	AB379742	3	AB437030	III	S
49767	Tongue (J)	0.5	<0.125	1	0.06	0.25	<0.03	AB379743	6	AB437034	XII	T
54349	Sputum (J)	0.5	<0.125	0.25	0.03	0.06	<0.03	AB379751	8	AB437039	I	U
54381	Sputum (J)	0.5	<0.125	0.125	0.03	0.125	<0.03	AB379752	14	AB437040	V	V
54604	Clinical isolate (TW)	0.5	1	1	0.125	0.25	<0.03	AB379754	16	AB437041	XII	W
55046	Clinical isolate (J)	0.5	<0.125	0.125	0.03	0.06	<0.03	AB379756	12	AB437043	VII	X

S: collected at August 2006, W: collected at February 2007.

J: Japan, USA: the United States of America, TW: Taiwan.

A, B, C and D indicated at the environmental isolate indicated the water supply system.

AMPH-B: amphotericin B, 5-FC: flucytosine, FLCZ: fluconazole, ITZ: itraconazole, MCZ: miconazole, MCFG: micafungin:

*; resistant, and ^+^; dose-dependent susceptibility based on CLSI M27-A2 protocol [42].

**Table 6:** Antifungal susceptibility and genotypes of *C*. *tropicalis* isolates.

		Susceptibility to antifungal drugs						MDR 1		ITSrRNA		Combined
IFM No.	Animal No.	AMPH-B	5-FC	FLCZ	ITZ	MCZ	MCFG	Accession No.	Genotype (1–11)	Accession No.	Genotype (I–V)	Genotype (A–M)
Dolphin isolates												
55217	No. 1 (S)	0.25	<0.125	>64*	>8*	0.5	<0.03	AB379757	1	AB437044	I	A
55220	No. 2 (S)	0.25	<0.125	2	0.125	1	<0.03	AB379759	1	AB437046	I	A
55373	No. 2 (W)	0.5	<0.125	64*	4*	0.5	<0.03	AB379779	1	AB437066	I	A
55229	No. 5 (S)	0.25	<0.125	32^+^	8*	2	<0.03	AB379761	2	AB437048	II	B
55233	No. 7 (S)	0.25	<0.125	64*	4*	0.5	<0.03	AB379763	1	AB437050	I	A
55235	No. 7 (S)	0.5	<0.125	>64*	2*	1	<0.03	AB379765	3	AB437052	I	C
55269	No. 10 (S)	0.25	<0.125	>64*	>8*	0.5	0.06	AB379766	2	AB437053	II	B
55379	No. 10 (W)	0.5	<0.125	0.25	0.06	0.25	<0.03	AB379780	2	ND		ND
55277	No. 14 (S)	0.25	<0.125	64*	>8*	0.5	<0.03	AB379768	4	AB437055	I	D
55383	No. 14 (W)	0.25	<0.125	64*	2*	2	<0.03	AB379781	5	AB437067	I	E
Environmental isolates												

55294	Pool-No. 1 (A) (S)	0.5	<0.125	4	0.25^+^	1	<0.03	AB379770	6	AB437057	I	F
55297	Pool-No. 2 (A) (S)	0.5	<0.125	16^+^	1*	1	<0.03	AB379772	1	AB437059	I	A
55299	Pool-No. 3 (A) (S)	0.5	<0.125	1	0.25^+^	1	<0.03	AB379774	1	AB437061	I	A
55301	Pool-No. 5 (B) (S)	0.5	<0.125	0.25	0.06	0.25	<0.03	AB379775	2	AB437062	II	B
55303	Pool-No. 6 (B) (S)	0.5	<0.125	0.25	0.06	0.25	0.03	AB379777	2	AB437064	II	B
55318	Sea Water (D) (S)	0.5	<0.125	0.5	0.06	0.25	<0.03	AB379778	2	AB437065	II	B
Reference isolates												

5746	Clinical isolate (J)	1	<0.125	2	0.25^+^	0.5	<0.03	AB379783	7	AB437069	I	G
41420	Clinical isolate (J)*	0.5	<0.125	>64*	8*	0.5	<0.03	AB379785	1	AB437071	I	A
52008	Clinical isolate (J)*	0.5	<0.125	>64*	>8*	16	<0.03	AB379787	1	AB437073	I	A
52010	Clinical isolate (J)*	0.5	<0.125	>64*	>8*	8	<0.03	AB379788	8	AB437074	I	H
52013	Clinical isolate (J)*	0.5	<0.125	2	0.25^+^	0.25	<0.03	AB379790	9	AB437076	I	I
52938	Feline cystitis (J)	0.5	<0.125	1	0.06	0.5	<0.03	AB379791	10	AB437077	III	J
53910	Blood (J)	0.5	<0.125	0.5	0.03	0.125	<0.03	AB379794	8	AB437079	IV	K
54637	Pharynx (J)	0.5	<0.125	0.5	0.125	0.125	<0.03	AB379796	9	AB437081	I	I
54674	Pharynx (J)	0.5	0.125	2	0.125	0.5	<0.03	AB379797	8	AB437082	I	H
54675	Pharynx (J)	0.5	<0.125	1	0.125	0.25	<0.03	AB379798	9	AB437083	V	L
55049	Blood (J)	0.5	<0.125	0.5	0.25^+^	0.5	<0.03	AB379801	11	AB437085	I	M
55256	Ocular mycosis (J)	0.5	<0.125	>64*	2*	2	<0.03	AB379802	1	AB437086	I	A

S: collected at August 2006, W: collected at February 2007, J: Japan.

A, B, C and D indicated at the environmental isolate indicated the water supply system.

AMPH-B: amphotericin B, 5-FC: flucytosine, FLCZ: fluconazole, ITZ: itraconazole, MCZ: miconazole, MCFG: micafungin,

*; resistant, and ^+^; dose-dependent susceptibility based on CLSI M27-A2 protocol [42].
